# Direct Liquid Injection in Comprehensive Two‐Dimensional Gas Chromatography Hyphenated to Quadrupole Time‐of‐Flight Mass Spectrometry Quality Profiling of Commercial Whiskies

**DOI:** 10.1002/jssc.70490

**Published:** 2026-07-14

**Authors:** Brian R. van 't Veer, Sander Affourtit, Hans‐Gerd Janssen

**Affiliations:** ^1^ Da Vinci Laboratory Solutions Rotterdam the Netherlands; ^2^ Laboratory of Organic Chemistry Wageningen University & Research Wageningen the Netherlands; ^3^ Unilever Foods Innovation Centre Wageningen the Netherlands

**Keywords:** comprehensive two‐dimensional gas chromatography, direct liquid injection, food analysis, high‐resolution mass spectrometry, whisky

## Abstract

Luxury food products such as olive oil, wine, and whisky are highly vulnerable to fraudulent practices. Their chemical complexity, fortunately, provides numerous potential adulteration markers, but unfortunately also necessitates the use of powerful, comprehensive profiling approaches involving detailed methods for sample cleanup and analysis. One technique capable of both targeted marker analysis and untargeted profiling is comprehensive two‐dimensional gas chromatography coupled to high‐resolution mass spectrometry (GC×GC‐HRMS). However, as with other chromatographic methods, this technique typically requires substantial sample preparation to eliminate contaminants, which increases the risk of losing valuable information. To address this issue, a direct‐injection GC×GC‐HRMS technique was developed for comprehensive quality profiling of whiskies. Multiple injection parameters were optimized to deal with the direct injection of a large series of whiskies with a focus on data quality. Overall, carryover percentages of less than 1.5% were obtained, except for long‐chain linear acids, sugar‐related compounds, and lignin‐related compounds (carryover at most 7.1%, 11.4%, and 18%, respectively). The method demonstrated adequate sensitivity to identify numerous quality marker compounds. The developed method was applied to 59 whiskies, with some samples being measured in multiple sequences to assess method stability. Finally, chemometric analyses proved the data to be of good quality. By employing techniques such as principal component analysis, hierarchical clustering analysis, and partial least squares regression, valuable information was obtained, including insights into the preparation process and prize categorization.

## 1 Introduction

Understanding the chemical complexity of food products provides essential information for food safety and food quality assessment. Food authentication and food fraud studies are two more specific application fields with obvious links to both quality and safety that also strongly rely on detailed chemical analyses. As far as food fraud is concerned, obviously, expensive, luxury food products are vulnerable to fraudulent practices. This includes olive oil, chocolate, wine, ginger, and numerous other artisanal products. Aged spirits, and in particular whisky, are also an appealing product category for fraud due to the high price, but, paradoxically, also due to the complexity of their composition. The high chemical diversity, on the one hand, enhances the number of specific analytes for detecting adulteration; but on the other hand, it imposes great demands on the analytical methodologies required for reliable quality control and fraud identification.

A wide range of analytical techniques has been proposed for the authentication, discrimination, and quality control of whiskies, including Raman spectroscopy, nuclear magnetic resonance (NMR) spectroscopy, Fourier‐transform infrared spectroscopy (FT‐IR), gas‐ and liquid chromatography (GC and LC), either or not hyphenated to mass spectrometry (MS), etc. [1]. Interestingly, some spectroscopic techniques are even able to perform through‐the‐bottle analysis, making them perfect candidates for the authentication of unopened bottles [2]. A truly comprehensive and detailed profile of a whisky, however, can only be obtained using chromatographic techniques, where a small amount of sample is necessary to perform the analysis. Numerous chromatographic methods for the analysis of whisky have been published in scientific literature or by trade organizations [1, 3, 4, 5, 6, 7]. Nearly all these methods include a sample preparation step to remove non‐volatile compounds such as sugars or polyphenolic structures originating from aging in wood barrels. Unfortunately, all sample collection and preparation protocols inherently pose the risk of reducing or removing compounds that may offer valuable quality and authenticity information [8]. While minimal sample preparation is generally preferred to preserve the integrity of the sample, sufficient preparatory steps must be employed to ensure robust and reproducible analyses using the selected chromatographic method. Obviously, the sample preparation method selected should ideally be automated, fast, and green, while maintaining a high compound coverage and sensitivity. Novel sample preparation techniques—most notably those that can be automated—can reduce the time and labor substantially.

As previously noted, obtaining a high level of detail in recorded fingerprints is an essential requirement for chromatographic methods employed in quality control and fraud detection. From that perspective, the preferred analysis method is comprehensive two‐dimensional GC coupled to high‐resolution MS (GC×GC‐HRMS). By incorporating both an additional separation dimension and HRMS, GC×GC‐HRMS enables both highly sensitive targeted marker analysis and highly detailed untargeted whisky profiling. Ideally, this technique is used with minimal sample preparation. Direct injection of whisky, however, suffers from several potential difficulties, including the low volatility of water and the potential risk of inlet contamination. Despite its challenges, direct injection could be possible by careful selection of the injection conditions. Several research groups have already proposed strategies to deal with direct injections of relatively clean aqueous samples, e.g., employing a polar stationary phase or using solvent venting or backflushing of the column [9, 10, 11, 12, 13, 14]. Application of such methods to samples rich in non‐volatiles so far has not been studied. We believe that, if carefully optimized, similar direct injection methods might also be applicable to more difficult samples such as whisky.

Since food‐fraud analysis typically requires the use of profiling methods combined with advanced unsupervised and supervised data processing methods such as principal component analysis (PCA) or partial least‐squares discriminant analysis (PLS‐DA) [8, 15, 16, 17, 18, 19], high requirements are imposed on data quality and stability. If sample preparation is to be eliminated, careful checks are needed to see if the data stability that is required for chemometric analysis can still be obtained.

The aim of this research was to develop a rapid, direct‐injection GC×GC‐Quadrupole Time‐of‐Flight (GC×GC‐QTOF) method for whisky quality profiling. Several method aspects were optimized to deal with the direct injection of a large series of whiskies without major instrumental modifications. Parameters studied include injection volume, split ratio, inlet temperature, and liner design. The developed method was evaluated for data quality through retention time and peak stability of control samples over time, and by applying chemometric analyses and correlation studies to differentiate whisky types and price categories.

## 2 Materials and Methods

### 2.1 Chemicals and Materials

**FIGURE 1:**
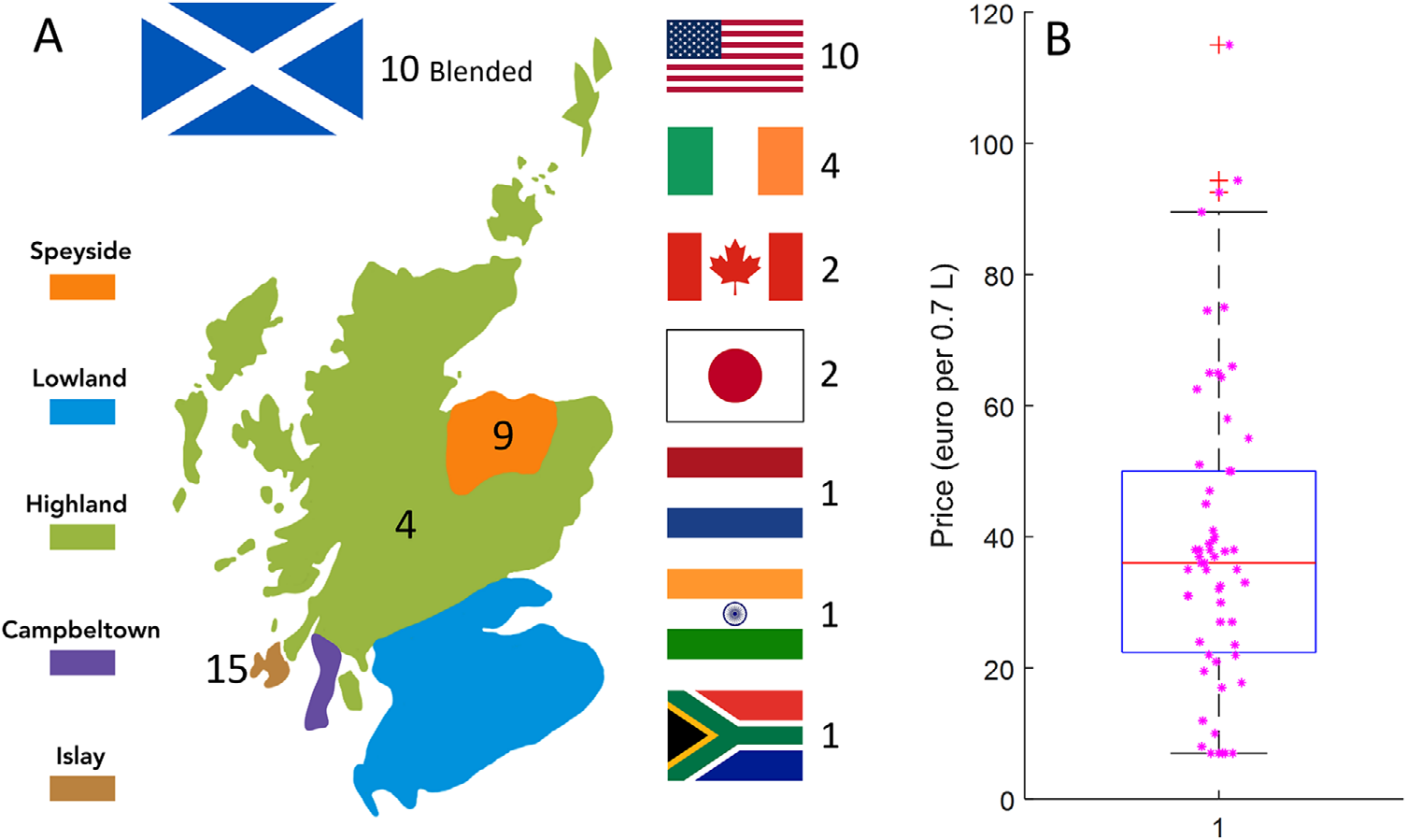
Origin of the whisky samples studied (A) and the overall price distribution (B). Region map adapted [23].

A diverse sample set consisting of 59 whiskies from different origins was studied. The distribution over the various countries and regions of origin is shown in Figure 1. The samples were either purchased online or obtained from liquor stores in the Netherlands or Germany and refrigerated until further use. To limit the price variability between liquor stores, multiple online sources were consulted, whereafter the lowest available price was reported (Table S1). Acetone was purchased from Biosolve (Valkenswaard, the Netherlands).

### 2.2 Instrumentation and Conditions

Initial experiments were performed on an Agilent 8890 GC (Agilent, Waldbronn, Germany), equipped with an Agilent 7650A automated liquid sampler and a Zoex ZX‐2 cryogen‐free modulator (Zoex, Houston, Texas). The instrument was fitted with an Agilent J&W DB‐5MS (30 m × 0.25 mm × 0.25 µm) as a ^1^D column and an Agilent J&W BPX‐50 (2.0 m × 0.10 mm × 0.10 µm) as a ^2^D column connected with a Trajan (Ringwood, Australia) SilTite μ‐Union. The oven temperature was set to an initial temperature of 70°C for 5 min. Next, it was programmed with a heating rate of 4°C/min to a final temperature of 300°C without a final isothermal hold (*t*_*method*_ = 62.5 min). A constant flow rate of helium carrier gas of 0.8 mL/min was used for all measurements, and the septum purge was kept at 3.0 mL/min. Modulation of the ^1^D effluent was performed with a modulation period of 4 s and a hot jet time of 0.35 s. The cold‐jet mass flow of the modulator was programmed to gradually decrease from 12 to 3.3 L/min at the end time to prevent poor reinjection of the heavier analytes. The hot jet temperature started at 250°C and was increased at 3°C/min to a final temperature of 400°C, where it was held until the end of the run. The transfer line temperature was set to 300°C. The inlet consisted of an Agilent Split/Splitless Inlet fitted with an Agilent Ultra Inert Liner (V = 870 µL). As part of the optimization, different masses of glass wool (Supelco, Darmstadt, Germany) were positioned at different heights in the liner.

Initial experiments employed an Agilent 5977C MSD as a detector, which was set to acquire mass spectra over an *m/z* range of 50–450 at an acquisition rate of 20 Hz. The ion source and quadrupole temperatures were 230 and 150°C, respectively. The electron energy was set to 70 eV, and the device was set to measure in positive mode. The injection was optimized based on a Box‐Behnken design with additional measurements of injection volumes in the range of 0.1–0.5 µL, split ratios of 25:1–100:1, and injector temperatures in the range of 150–350°C, totaling 18 conditions. Results were assessed based on chromatographic performance, peak intensities, and evidence of inlet/column overloading.

The peak‐intensity repeatability at the optimized settings was investigated by repeat injections (*n* = 11) and by evaluating the relative standard deviation (RSD) of marker‐peak intensities. The same repeatability experiment was repeated for the lower split ratio of 10:1 while keeping injection volume and inlet temperature fixed to their respective optimal values to investigate whether method sensitivity could be enhanced without sacrificing repeatability.

After method optimization, the method was transferred to a similar system now employing an Agilent QTOF 7250A MS/MS instrument, which was set to acquire single profile spectra over an *m/z* range of 50–500 at an acquisition rate of 50 Hz. Mass calibration was performed before each sequence. The solvent delay was set to 6.5 min to avoid detection of the high amounts of short‐chain linear and branched alcohols present in whisky. The whisky samples were analyzed in batches of approximately 20 samples over a time period of 4 weeks. Five blank measurements using acetone were performed at the start of a sequence with three cleanup blanks at the end. Each sample was measured three times in direct succession. After each triplicate measurement, a blank analysis was carried out to ensure the absence of carryover effects. A total sequence consisted of around 100 injections. After every sequence, the inlet septum was replaced, and the liner was rinsed with 2 mL of acetone to remove septum residues. This was done without removing the glass wool packing; hence, the glass wool plug was identical for all measurements. After the second batch, the first three samples of the first batch were measured again to monitor chromatographic performance in terms of retention‐time stability and peak widths.

Instrument control was performed using Agilent MassHunter software. Data analysis was performed using GC Image GC×GC‐HRMS software v2025r2 (Zoex) and the included Investigator program, and MATLAB R2025B (MathWorks, Natick, MA, United States). Chromatogram alignment and peak detection were automated using a feature template constructed by the built‐in algorithm [20]. The NIST 2023 Retention Index (RI) library and mass spectral library were used in the process of compound identification. RI calibration was established using the RI of the linear acids and ester‐like counterparts in combination with the NIST 2023 mass spectral library. Tentative compound identities were established based on a combination of library searches in the NIST 2023 mass spectral library, the calculated compound RI, and the chemical formula using exact mass. Sugar‐related compounds were only identified as such; full elucidation of the specific saccharide structure was beyond the scope of the current untargeted profiling method. Quantification of the compounds was based on the 100% method, where the percent response of a peak was defined as the integrated peak volume divided by the total image volume. This normalization allows for a better comparison between samples as opposed to absolute peak intensity or peak volume by correcting for small variations in, e.g., injection volumes and detector sensitivity. The QTOF detector's limited dynamic range may cause volume percentages to vary with injection mass, but it also helps to scale by reducing extreme compound intensities.

Since whisky adulteration often happens by diluting whisky with cheaper alternatives or by mislabeling a product as belonging to a protected name, simulating a forged whisky is straightforward. In this research, we have opted to simulate a fraudulent mixture of the relatively expensive Glenfiddich 18 with one of the cheapest whiskies in the dataset (i.e., Dean's Blended Scotch Whisky) by adding the chromatograms and mass spectra together in a 1:3 ratio, respectively. Next, the chromatogram was averaged, yielding a dilution factor of 4. After formal data analysis, this adulterated chromatogram was treated as a novel sample and subjected to the data analysis workflow.

## 3 Results and Discussion

GC analysis of aqueous samples high in nonvolatile species, such as sugars or colorants, poses several risks. These include a poor method robustness with shifts in retention time due to the buildup of non‐volatile species on the column, an increased background signal, and column degradation due to water vapor. Another challenge of injecting aqueous samples in GC is backflash of solvent into the GC carrier gas inlet due to the rapid volume expansion caused by the flash vaporization. This backflash may contribute to extensive carryover effects, severely impacting subsequent measurements. As mentioned in the introduction, careful selection of the injection parameters is essential to prevent these issues and allow direct injection without compromising method performance. Optimization of the injection setting, unfortunately, is not trivial because many factors are interrelated and different method aspects result in contradictory requirements. Increasing the liner volume, for example, is a straightforward manner to permit higher injection volumes without risking backflash, but a larger liner volume may broaden injection bands, adversely affecting chromatographic performance. With regard to sensitivity, higher injection volumes may seem favorable. However, besides the risk of backflash or column overloading, higher inlet temperatures may be required to effectively vaporize the increased sample amount, given the very high heat of evaporation of water. These higher inlet temperatures, in turn, may lead to an increased risk of backflash and an increased possibility of thermal reactions of compounds in the sample. Sugars, present in more or less all whisky samples due to the fermentation and cask maturation steps, can caramelize or char at high temperatures, contaminating the liner and producing new volatiles not found in the original sample [21, 22]. Parameter selection is therefore critical. The liner should retain non‐GC‐amenable compounds like sugars and colorants, but while present in the injector, this material should not result in artifact formation during the analysis sequence.

**FIGURE 2:**
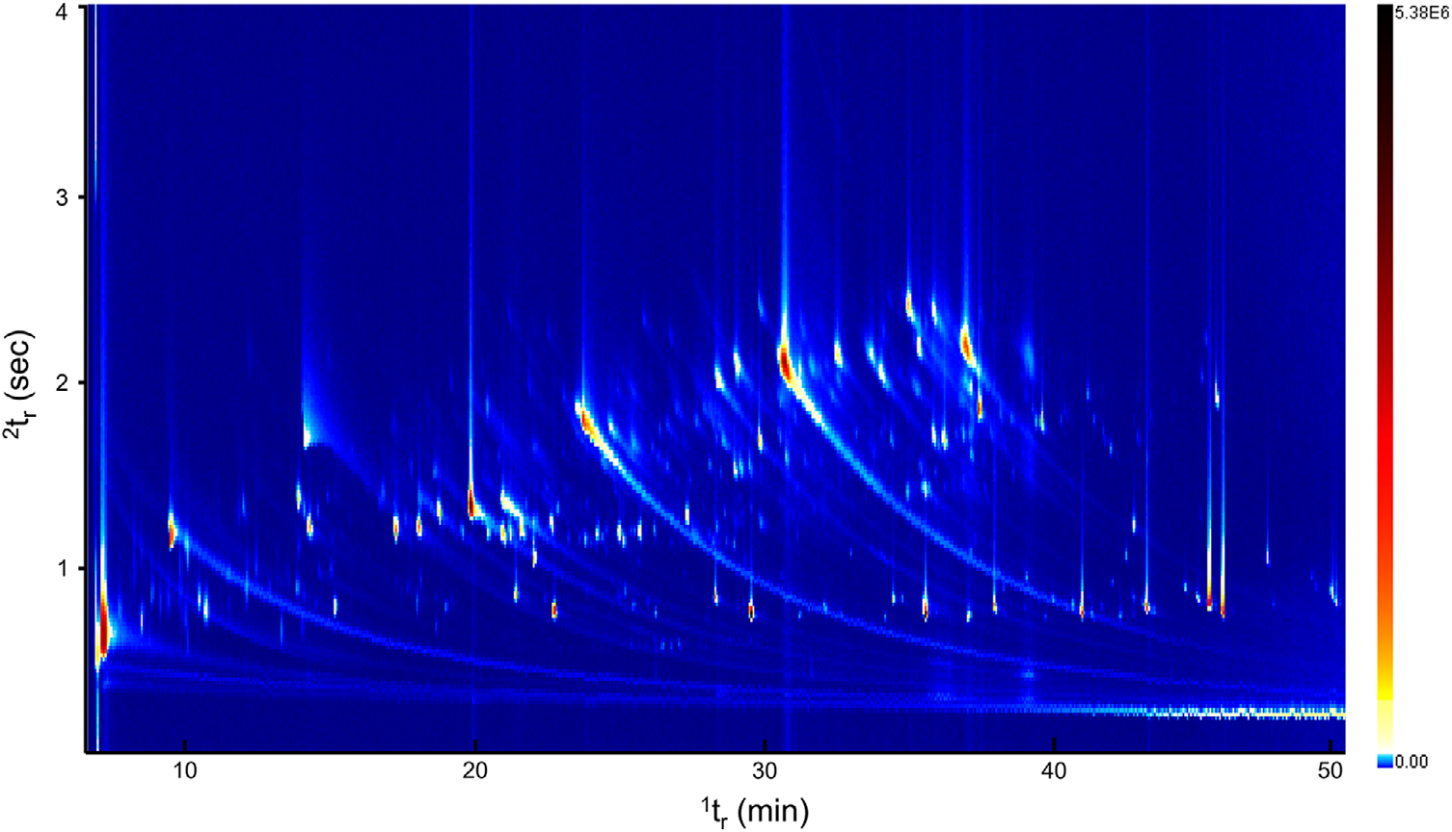
Chromatogram of the Laphroaig PX whisky as measured with the optimized settings.

**FIGURE 3:**
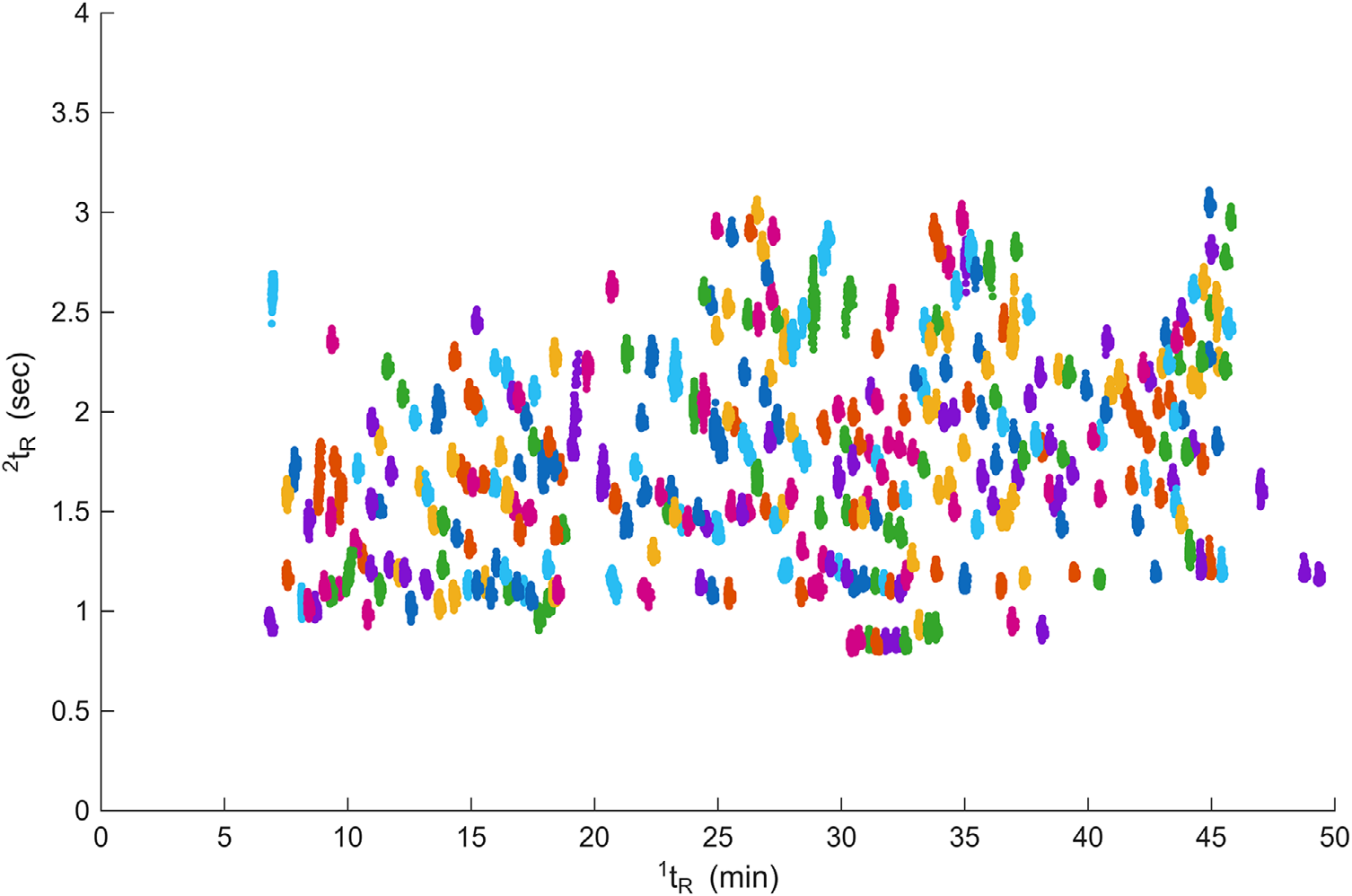
Retention‐time repeatability for all peaks in all sample measurements.

In the optimization, several factors were studied. Overall, a conservative approach was taken by choosing parameter values that carry a low risk of backflash or column overloading and gradually move towards higher injected amounts. The injector temperature was chosen based on three key considerations: ensuring effective transfer of heavy analytes to the column, reducing material accumulation within the liner, and preventing excessive caramelization reactions. The inlet temperature was varied from 150 to 350°C, and the injection volume was varied between 0.1 and 0.5 µL. The split ratio was studied between 100:1 and 25:1. The liner volume was fixed, and all experiments were performed with a single‐taper, low‐pressure‐drop liner. Different amounts of glass wool packings were evaluated, and also the position of the glass wool plug relative to the syringe outlet was varied. Although lowering the split ratio could further improve the sensitivity of the method, it will also result in severe overloading of the ^1^D column with the main whisky ingredients. Moreover, lower split ratios will also increase the amount of bleeding from the liner. The results of the optimization are summarized in Table 1. Experiments with inlet temperatures of 350°C showed an increased baseline due to septum bleed, negatively impacting the SNR. Likewise, compound intensities and the number of detected compounds present in the chromatogram did not increase when using the elevated injection temperature of 350°C as opposed to 250°C. Conversely, an inlet temperature of 150°C decreased both compound intensities and compound diversity, indicating incomplete sample transfer. The same results were obtained when lowering injection volumes, which has the additional drawback of a poorer injection‐volume repeatability. Therefore, an inlet temperature of 250°C and an injection volume of 0.5 µL were used for the repeatability experiments. Herein, the optimal split ratio of 25:1 was further decreased to 10:1 to investigate whether the sensitivity could be increased. Repeated measurements (*n* = 11) were performed for each of these split ratios. Comparing the RSD distributions for the percent response, peak intensity, and the peak volume showed that the percent response yielded the lowest RSD values and was thus most repeatable (Figure S1). Conversely, lowering the split ratio to 10:1 led to less stable injections, as was seen by the higher RSD distribution. Therefore, a split ratio of 25:1 was used for further experiments. In combination with the inlet temperature of 250°C and an injection volume of 0.5 µL, this resulted in chromatograms similar to those shown in Figure 2. Due to the inherent ordering of peaks in the GC×GC chromatograms, relatively nonpolar and volatile compounds elute in the lower early region of the chromatogram, whereas more polar and larger compounds, such as sugars, have more retention and thus elute later and in the upper region. Since issues with artifact formation or carryover can never be fully ruled out, a blank sample was measured immediately after a whisky triplicate. Additionally, several samples were re‐measured over different sequences analyzed on different days. This was also done after inlet maintenance to assess the stability of the method. In Figure 3, the retention times of all the aligned peaks are shown for all 204 sample measurements. Points that derive from the same peak have the same color in that region. Overall, the retention time distribution demonstrates consistency across the majority of peaks. Higher retention‐time deviations are observed for peaks with longer first‐ and second‐dimension retention times. Typically, retention time variability in the first dimension was less than 1% for all compounds and was less than approximately 2.3% in the second dimension for 95% of the compounds. Comparing the retention times of some common whisky compounds present in all samples, such as the linear saturated fatty acids and their ethyl esters, showed no significant retention shifts over the analysis series, indicating the absence of excessive column degradation. Also, the background signal corresponding to the column bleeding did not increase significantly for later measurements, further demonstrating the absence of stationary phase degradation.

Carryover could be another issue that might arise. Since it is likely to be most prominent for highly concentrated peaks, it was established using the sample with the highest overall intensities (i.e., Laphroaig PX). An overview of the compounds that were monitored and their carryover is given in Table S2. In general, carryover was found to be less than 1.5% for short‐chain alcohols, ethyl esters, and typical phenolic compounds. Contrarily, longer‐chain alcohols and acids gave carryover percentages typically around 2.8% and at most 7.1%. Carryover for sugar‐like compounds could be as high as 11.4%. However, in our case, such high sugar amounts were only present in one whisky, and all monitored intensities returned to less than 1% carryover after the fifth blank measurement. For lignin‐related compounds, carryover percentages ranged from 0.1% for guaiacol to 18% for sinapyl alcohol. When measuring whiskies with such high amounts, extra care should be taken, and it could be recommended to insert multiple rapid blank runs between different samples.

Another issue that can occur is peak tailing resulting from column contamination or from the formation of active sites on the stationary phase or column wall. Tailing can negatively affect data quality, for example, due to incorrect peak integration. Especially whiskies where non‐volatile colorant has been added, or those that have been matured on casks rich in sugars, suffer from this risk. These non‐volatile compounds may display incomplete vaporization and contaminate the liner. Visual inspection of the liner indeed showed a small, brown/black local injection spot in the quartz wool (Figure S2). Fortunately, the acceptable carryover results showed that the observed residue had no negative impacts.

**FIGURE 4:**
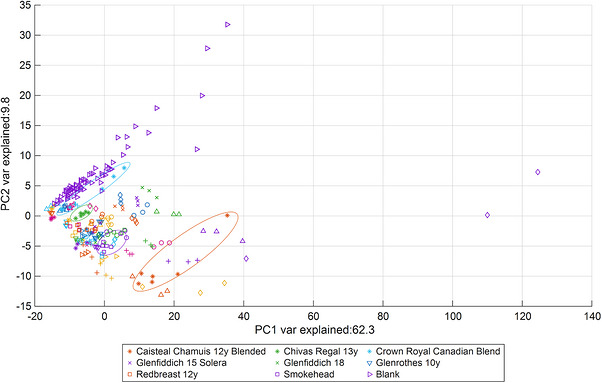
Principal component analysis of the measured whiskies and the blank measurements. The samples in the legend have been measured over multiple sequences. A full overview of legend entries is included in Figure S4.

As mentioned in the introduction, a high data quality is essential for subsequent chemometric analysis of the data sets recorded. While run‐to‐run variability can be minimized by robust method development, factors such as detector drift are more difficult to control. To enhance data consistency and reduce systematic bias, standard preprocessing techniques such as chromatogram alignment and data normalization were implemented using the appropriate features in the GC Image software. Similar to what has been described earlier, these steps provided significantly lower RSD values for the percent response as opposed to the peak value or integrated volume (Figure S3). As can be expected, this effect was more pronounced for the QC samples, since that data is more influenced by factors such as detector drift. When comparing the percent response RSDs, the mean and median intra‐day RSDs were 7.5% and 5.6%, respectively. The mean and median inter‐day RSDs were significantly higher at 17.5% and 14.5%, respectively. The distribution of RSD values is shown in Figure S3. Since data quality is especially critical in unsupervised data interrogation methods such as PCA, a PCA plot was generated for the entire sample set of 59 samples, including the blank samples and the samples that had been measured multiple times. Besides z‐scoring, no further data preprocessing was performed to keep the PCA purely exploratory for all detected peaks. The resulting plot is shown in Figure 4 (for the full overview of legend entries, see Figure S4). In this figure, the blank measurements are denoted by the purple triangles and are clustered in the top left by the PCA. Clustering of the triplicate measurements can also be observed, with the triplicate measurements ending up either in the same region or in the same direction from the blanks. Several samples that have been measured over different sequences are outlined in the figure with their respective color. Even for most of these samples, good clustering is still observed, confirming a good retention time stability. Clearly, the PCA method can handle the slight day‐to‐day differences in our data, or phrased differently, the data stability of our direct injection method is sufficient to allow the use of even the most critical unsupervised methods, such as PCA. Admittedly, a standard data alignment step was applied in the GC image software, so small variations might still have been present.

From the PCA plot, a number of interesting conclusions regarding whisky quality can be drawn. As an example, some whiskies are close to the blanks. These whiskies are almost exclusively those at the cheaper end of the price distribution (Figure 4, left). Indeed, the cheaper whiskies show an overall low intensity across all compounds. Dilution with water, bringing the alcohol content down to 40%, is an allowed method to make whisky accessible to a broader range of consumers. This results in simpler chromatograms and, generally, a lower price. Whiskies that are bottled without any dilution (i.e., cask strength) are often on the more expensive side. In addition to dilution, shorter maturation times are obviously favorable from a commercial point of view. This results in fewer compounds extracted from the wood cask and again simpler chromatograms. Contrarily, longer maturation times and cask finishes may contribute to the compound diversity and concentration differences. On the right of the PCA plot in Figure 4, two datapoints of the Laphroaig PX whisky are plotted. This was the whisky that contained the highest overall peak intensities within the chromatogram.

**FIGURE 5:**
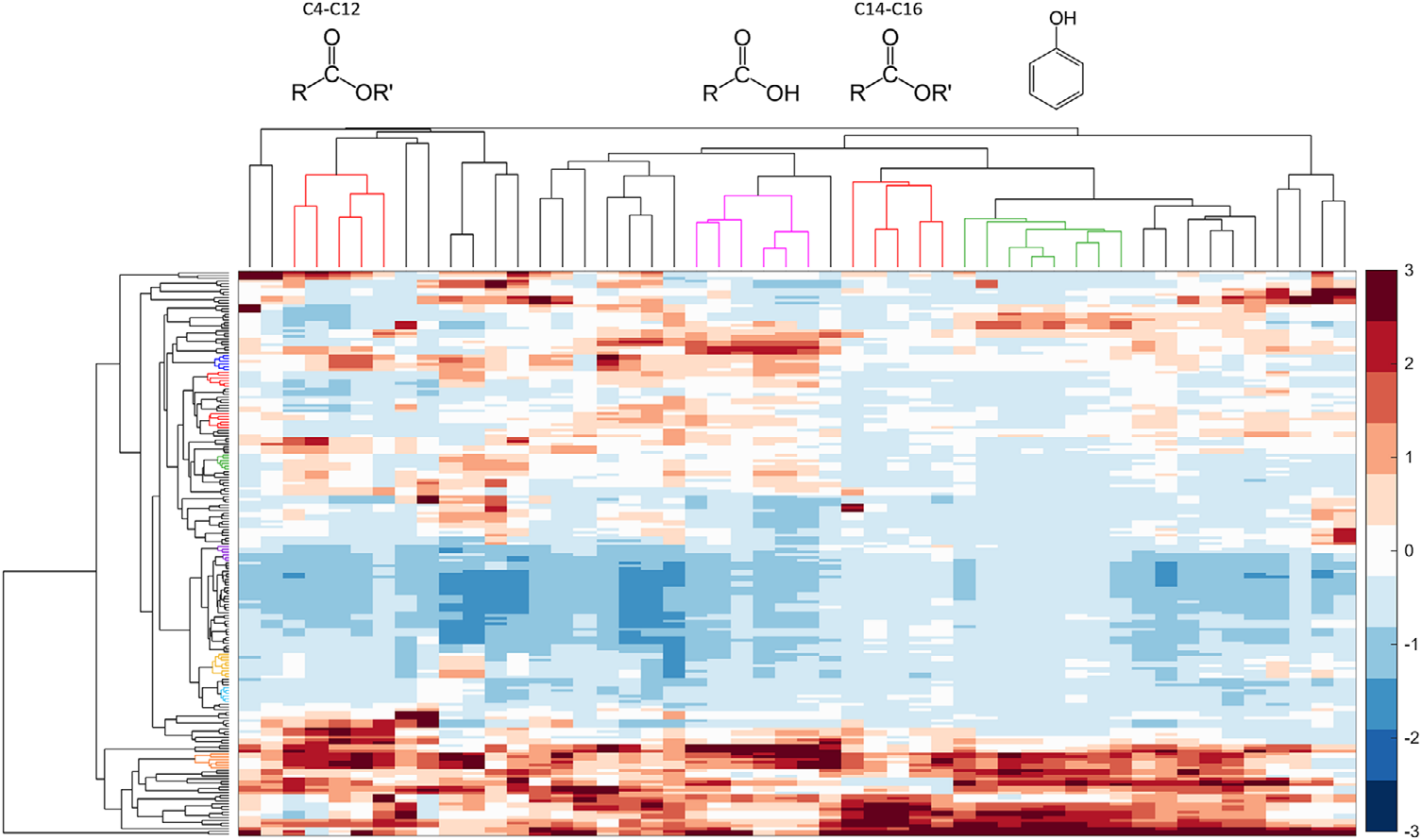
Hierarchical clustering analysis of 59 whiskies based on the occurrence of 50 key compounds. The individual whisky measurements are clustered vertically, the compounds horizontally. Measurements from multiple sequences have been color‐coded depending on the control samples. The compounds have been color‐coded depending on compound group, from left to right: short‐chain ethyl esters in red; linear acids in pink; long‐chain ethyl esters in red; phenolic compounds in green.

A second widely used unsupervised data analysis tool is hierarchical clustering analysis (HCA). HCA detects groups of samples based on the occurrence of common compounds. Such similarities in composition can provide information, e.g., on similarities in the processing method. By including the control sample measurements (i.e., the same sample measured in multiple sequences) in the HCA, the repeatability and batch consistency can be evaluated, since obviously these analyses are expected to cluster. To filter out incorrectly assigned peaks and increase repeatability, the Fisher Ratio calculated by the GC Image software was used with the technical replicates as the grouping factor. The result of the HCA is shown in Figure 5 using the 50 compounds with the highest F‐values. The vertical direction of the graph shows the clustering of the 204 measurements (i.e., 59 samples in triplicate with additional control sample measurements), whereas the horizontal dimension shows the clustering of different compounds. Triplicate measurements show excellent clustering, as shown by the very small width of the triplicate clusters. Moreover, measurements of the same sample in different sequences show up as one cluster, e.g., the clusters in green, purple, orange, and blue. The clusters on the left that are colored red also derive from the same samples. Unfortunately, these two triplicate measurements are not clustered correctly. This may be explained by their mostly low intensities, meaning that small changes in the background signal may have a bigger impact on the data. The bottom samples displayed in Figure 5 show an overall high compound intensity, denoted by their red color. The compounds that are mainly responsible for the clustering of the bottom samples are phenolic in nature. Most likely, these result from the peat smoke used for heating and drying the malted barley. Finally, it is known that the type of cask used for maturation also plays an important role in flavor creation. This too becomes evident from the samples in the HCA, revealing grouping based on sherry cask finishes. In these samples, comparatively elevated sugar levels were observed alongside a greater compound diversity and intensity. This all together underlines the fact that the direct injection method is reliable over multiple sequences and for samples measured at different timepoints. Similar to the PCA, clustering is also observed for whiskies at the cheaper end of the distribution, as can be seen from the low overall intensities for these samples, denoted by the blue colors. Clearly, the HCA plot highlights useful production insights and shows that our direct‐injection method yields data of sufficient quality for reliable interpretation, even when using critical, unsupervised chemometric methods such as PCA and HCA.

**FIGURE 6:**
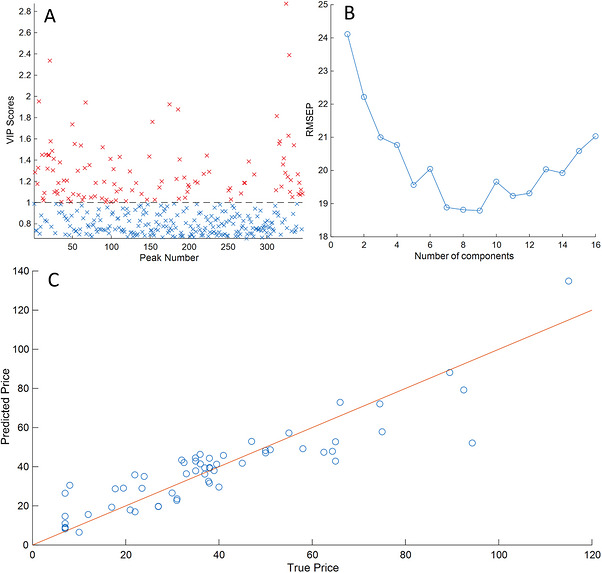
Partial least squares model overview for a model deriving whisky price from the chemical composition, with (A) variable importance for prediction score plot; (B) root mean squared error of prediction for different numbers of components; (C) distribution of model‐predictions and true values for a model with 5 components, line added for visual clarity.

A final application of the data set collected here is given below, where the commercial value of a whisky was modeled as a function of the compound intensities. This was done using PLS regression. In Figure 6, the main results from the model construction and validation are summarized. Figure 6A shows the “variable importance for prediction (VIP)” scores that are associated with PLS. VIP scores are commonly used in PLS for variable selection by selecting those that have a score of 1 or higher, as shown by the red datapoints in Figure 6A. Among the compounds identified, the primary acids and esters were recognized as particularly significant indicators of price. From a cask maturation perspective, this seems logical since a longer maturation time allows for increased leaching of compounds from the cask into the whisky, while longer maturation also makes the whisky more expensive. In Figure 6B, the root mean squared error of prediction (RMSEP) is shown for different numbers of components. Because the model is unlikely to improve and more inclined to overfit to our data if more components are included, five components were chosen as optimal. Application of the model to the data yields the graph shown in Figure 6C, where the true and predicted whisky prices are shown. Although some deviations in the true versus predicted prices exist, the method outlined above was effective in identifying compounds that are key in determining the whisky price category. As a proof of principle, the simulated adulterated chromatogram was subjected to the data analysis workflow, encompassing data import, peak detection, alignment, and overlaying the created feature template to identify marker compounds. Subsequently, the resulting percentage‐response table was projected onto the PCA axes of a PCA excluding the blank measurements (Figure S5). As expected, the simulated adulteration grouped together with the cheaper whiskies, moving away from the more expensive Glenfiddich 18 and towards the Dean's Blended Scotch Whisky. In a similar manner, the PLS regression model was applied to the simulated adulteration, resulting in a predicted price point of approximately €18.86. This is in line with the expectation that dilution and other adulterations negatively affect true and perceived quality, demonstrating the method's potential for adulteration detection.

## 4 Conclusion

A direct‐injection GC×GC‐QTOF method was successfully developed, allowing for repetitive direct injections of whiskies while maintaining method robustness. Carryover results were acceptable, with typical carryovers being less than 2.8%, except for long‐chain acids and sugar‐ and lignin‐related compounds. PCA of all obtained data showed clear differences and similarities for the blank and multiplicate measurements (i.e., both in direct succession and at different measurement times). Data were shown to be of good quality for further chemometric analysis using both supervised and unsupervised methods, such as HCA and PLS. Application of the method to a case study involving the quality profiling of 59 whiskies was successful in identifying key compounds responsible for making up the type and price category of the whiskies, clearly demonstrating its potential in mitigating extensive sample preparation procedures.

## Author Contributions

**Hans‐Gerd Janssen**: conceptualization, supervision, project administration, and writing – review and editing. **Brian R. van ‘t Veer**: conceptualization, investigation, writing – original draft, methodology, project administration, formal analysis, and visualization. **Sander Affourtit**: resources, supervision, and writing – review and editing.

## Funding

This research received no specific grant from any funding agency in the public, commercial, or not‐for‐profit sectors.

## Conflicts of Interest

Brian van ‘t Veer and Sander Affourtit are employed by Da Vinci Laboratory Solutions, an instrument company focusing on the development of chromatographic analyzers. Hans‐Gerd Janssen is employed by Unilever, a multinational company active in food, home, and personal care products. The authors declare no other conflicts of interest.

## Declaration of Use of Artificial Intelligence‐assisted Technologies

During the preparation of this work, the authors used GPT‐5.2 in order to improve the readability and language of the work. After using this tool/service, the authors reviewed and edited the content as needed and take full responsibility for the content of the published article.

5

**TABLE 1:** Overview of method parameter values evaluated for inlet temperature (T_inlet_), injection volume (V_inj_), and split ratio, with corresponding performance criteria. SNR: Signal‐to‐noise ratio; n_peaks, SNR > 3_ is the number of peaks above a signal‐to‐noise threshold of 3; 5HMF: Abbreviation used for 5‐hydroxymethylfurfural; w_i_: Peak width where i corresponds to the chromatographic dimension.

Split ratio	T_inlet_ (°C)	V_inj_ (µL)	n_peaks, SNR > 3_	SNR C6‐C16 esters	SNR 5HMF	Total average SNR	w_1_ (s)	w_2_ (s)	Skew_1_ mean	Skew_2_ mean
50:1	250	0.5	527	185	66	8	0.394	0.426	0.063	0.025
50:1	350	0.5	719	193	44	24	0.394	0.397	0.073	0.097
25:1	150	0.5	477	321	7	9	0.401	0.437	0.051	0.023
25:1	350	0.5	743	569	91	30	0.387	0.391	0.095	0.128
100:1	150	0.5	332	61	3	4	0.410	0.452	0.042	−0.005
100:1	250	0.5	380	83	20	6	0.402	0.438	0.060	0.014
100:1	350	0.5	663	94	16	19	0.398	0.406	0.066	0.091
25:1	250	0.5	757	761	282	21	0.387	0.420	0.086	0.014
100:1	250	0.1	415	17	4	4	0.412	0.445	0.031	0.021
25:1	250	0.1	443	96	31	6	0.402	0.439	0.058	0.017
25:1	150	0.1	318	8	3	3	0.412	0.453	0.033	0.008
25:1	350	0.1	755	92	32	22	0.401	0.404	0.046	0.076
25:1	150	0.3	496	200	6	8	0.405	0.443	0.048	0.002
25:1	250	0.3	489	328	76	13	0.337	0.374	0.086	0.053
25:1	350	0.3	637	49	14	5	0.410	0.443	0.032	−0.046
50:1	350	0.3	718	162	51	25	0.395	0.395	0.070	0.128
50:1	250	0.3	462	114	25	7	0.400	0.435	0.052	0.035
50:1	150	0.5	393	151	4	6	0.403	0.442	0.040	0.015

## Supporting information




**Supporting File**: jssc70490‐sup‐0001‐SuppMat.docx.

## Data Availability

The data supporting this study's findings are available from the corresponding author upon reasonable request.
