# Supplementary material for: Direct Liquid Injection in Comprehensive Two‐Dimensional Gas Chromatography Hyphenated to Quadrupole Time‐of‐Flight Mass Spectrometry Quality Profiling of Commercial Whiskies
Source: J Sep Sci. 2026 Jul 14;49(7):e70490. doi: 10.1002/jssc.70490 (PMC13367185; doi:10.1002/jssc.70490)
Supplement: Supplementary file 1 — Supporting File: jssc70490‐sup‐0001‐SuppMat.docx. [file JSSC-49-e70490-s001.docx]

Supplementary Material

Direct liquid injection in comprehensive two-dimensional gas chromatography hyphenated to quadrupole time-of-flight mass spectrometry quality profiling of commercial whiskies

Brian R. van ‘t Veer^a,b,*^, Sander Affourtit^a^, Hans-Gerd Janssen^b,c^

^a^: Da Vinci Laboratory Solutions, Sydneystraat 5, 3047 BP, Rotterdam, The Netherlands

^b^: Laboratory of Organic Chemistry, Wageningen University & Research, Stippeneng 4, 6708 WE, Wageningen, The Netherlands

^c^: Unilever Foods Innovation Centre, Bronland 14, 6708 WH, Wageningen, The Netherlands

Contents

[Supplementary table 1 – Sample information 3](#_Toc232615119)

[Supplementary figure 1 – Repeatability experiments 5](#_Toc232615120)

[Supplementary table 2 – Carryover results 6](#_Toc232615121)

[Supplementary figure 2 – Liner Inspection 8](#_Toc232615122)

[Supplementary figure 3 – RSD overview for all samples 9](#_Toc232615123)

[Supplementary figure 4 – PCA including blank measurements 10](#_Toc232615124)

[Supplementary figure 5 – Samples PCA including the simulated adulteration 11](#_Toc232615125)

## Supplementary table 1 – Sample information

*Table S1: Full dataset of 59 whiskies including the price, origin, cask, and maturation age information.*

| **Name** | **Price (0.7)** | **Region** | **Oak** | **Oak_American** | **Oak_European** | **Oak_Charred** | **Bourbon** | **Sherry** | **Oloroso** | **PX** | **Maturation Years** |
| --- | --- | --- | --- | --- | --- | --- | --- | --- | --- | --- | --- |
| Aberlour 12 | 38 | Speyside |  |  |  |  | 1 | 1 |  |  | 12 |
| Amrut | 36 | Indian | 1 | 1 |  |  |  |  |  |  | NaN |
| Ardbeg 10 | 47 | Islay |  |  |  |  | 1 |  |  |  | 10 |
| Ardbeg An Oa | 65.99 | Islay | 1 |  | 1 | 1 | 1 |  |  |  | NaN |
| Ardbeg Wee Beastie | 39.5 | Islay |  |  |  |  |  |  |  |  | 5 |
| Bains Cape Mountain whisky | 26.99 | South African |  |  |  |  | 1 |  |  |  | 3 |
| Big Peat | 36.95 | Islay Blend |  |  |  |  |  |  |  |  | NaN |
| Bourbon Elijah Craig Small Batch | 35.95 | Bourbon | 1 |  |  |  |  |  |  |  | 8 |
| Bourbon Evan Williams Black | 19.5 | Bourbon |  |  |  |  |  |  |  |  | NaN |
| Bourbon Larceny Small Batch | 38.95 | Bourbon | 1 |  |  |  |  |  |  |  | 6 |
| Bowmore 15 | 62.5 | Islay |  |  |  |  | 1 | 1 | 1 |  | 15 |
| Bowmore Legend | 30.95 | Islay |  |  |  |  | 1 |  |  |  | NaN |
| Bulleit bourbon | 33 | Bourbon | 1 | 1 |  | 1 |  |  |  |  | NaN |
| Bunnahabhain | 36.95 | Islay |  |  |  |  | 1 | 1 |  |  | 12 |
| Bushmills Black Bush Caviste | 23.49 | Irish |  |  |  |  | 1 | 1 |  |  | NaN |
| Caisteal Chamuis 12y Blended | 55 | Islay Blend |  |  |  |  |  | 1 | 1 |  | 12 |
| Canadian whisky | 10 | Canadian |  |  |  |  |  |  |  |  | NaN |
| Cardhu 12 | 37.99 | Speyside | 1 |  |  |  |  |  |  |  | 12 |
| Chivas Regal 13y | 37.95 | Blend |  |  |  |  |  | 1 | 1 |  | 13 |
| Crown Royal Canadian Blend | 20.95 | Canadian | 1 |  |  |  |  |  |  |  | NaN |
| Deans Blended Whisky | 6.99 | Blend |  |  |  |  |  |  |  |  | NaN |
| Finlaggan Old Reserve | 21.95 | Islay |  |  |  |  |  |  |  |  | NaN |
| Glen Moray Elgin Classic | 17.75 | Speyside |  |  |  |  | 1 |  |  |  | 7.5 |
| Glen Talloch Blend Choice | 16.99 | Blend | 1 |  |  |  |  |  |  |  | 5 |
| Glenfiddich 15 Solera | 50 | Speyside | 1 |  |  |  | 1 | 1 | 1 |  | 15 |
| Glenfiddich 18 | 93 | Speyside |  |  |  |  | 1 | 1 | 1 |  | 18 |
| Glenlivet 15 french oak reserve | 64.99 | Speyside | 1 |  | 1 |  |  | 1 |  |  | 15 |
| Glenlivet Captains Reserve | 40.95 | Speyside |  |  |  |  | 1 | 1 |  |  | 10 |
| Glenrothes 10y | 37.95 | Speyside | 1 |  |  |  |  | 1 |  |  | 10 |
| Golden Dew Blended Scotch Whisky | 6.99 | Blend |  |  |  |  |  |  |  |  | 3 |
| Highland Park 12 | 32.5 | Highland | 1 |  |  |  |  | 1 |  |  | 12 |
| Highland park Valknut | 58 | Highland |  |  |  |  |  |  |  |  | NaN |
| Jack daniels | 22 | Tennessee Whisky |  |  |  |  | 1 |  |  |  | NaN |
| Jameson | 26.99 | Irish | 1 |  |  |  |  |  |  |  | 4 |
| Jameson black barrell | 31 | Irish |  |  |  | 1 | 1 | 1 |  |  | NaN |
| Johnny Walker Black Label | 23.95 | Blend |  |  |  |  |  |  |  |  | NaN |
| Johnny Walker Green Label | 34.95 | Blend |  |  |  |  |  |  |  |  | NaN |
| Lagavulin 16y Distillers Edition (2000-2016) | 115 | Islay | 1 | 1 | 1 |  |  | 1 |  | 1 | 16 |
| Laphroaig PX | 52.15 | Islay | 1 |  |  |  | 1 |  |  | 1 | NaN |
| Laphroaig Select | 29.95 | Islay |  |  |  |  | 1 | 1 | 1 |  | NaN |
| Macallan Gold Double Cask | 64.99 | Speyside | 1 | 1 | 1 |  |  | 1 | 1 |  | NaN |
| McGlennon blended scotch whisky | 6.99 | Blend |  |  |  |  |  |  |  |  | NaN |
| Mic Mac blended whisky | 6.99 | Blend |  |  |  |  |  |  |  |  | NaN |
| Millstone Peated Oloroso (Gall&Gall Edition 2018) | 49.99 | The Netherlands |  |  |  |  |  | 1 | 1 |  | 5 |
| Nikka from the barrel | 64.33 | Japanese Blend | 1 | 1 |  |  |  |  |  |  | NaN |
| Oban Bay Reserve Game of Thrones | 89.5 | Highland |  |  |  |  |  |  |  |  | NaN |
| Oban Distillers Edition | 92.5 | Highland | 1 | 1 |  |  |  | 1 |  | 1 | NaN |
| Old style american whisky | 7 | American Whisky |  |  |  |  |  |  |  |  | NaN |
| Queen Margot 3y | 6.99 | Blend |  |  |  |  |  |  |  |  | 3 |
| Queen Margot 8y | 11.95 | Blend |  |  |  |  |  |  |  |  | 8 |
| Redbreast 12y | 45 | Irish |  |  |  |  | 1 | 1 | 1 |  | 12 |
| Rittenhouse rye | 35 | Rye Whisky |  |  |  |  |  |  |  |  | 4 |
| Smokehead | 32 | Islay | 1 |  |  |  |  |  |  |  | NaN |
| Talisker Dark Storm | 37.765 | Islay | 1 |  |  | 1 |  |  |  |  | NaN |
| Talisker skye | 35 | Islay |  |  |  |  |  |  |  |  | NaN |
| Toki suntory | 40 | Japanese Blend |  |  |  |  |  |  |  |  | NaN |
| Western gold 5 | 8 | Bourbon |  |  |  |  |  |  |  |  | 5 |
| Whistlepig 10 | 75 | Rye Whisky |  |  |  |  |  |  |  |  | 10 |
| Woodford Reserve | 51 | Bourbon | 1 |  |  | 1 |  |  |  |  | NaN |

## Supplementary figure 1 – Repeatability experiments


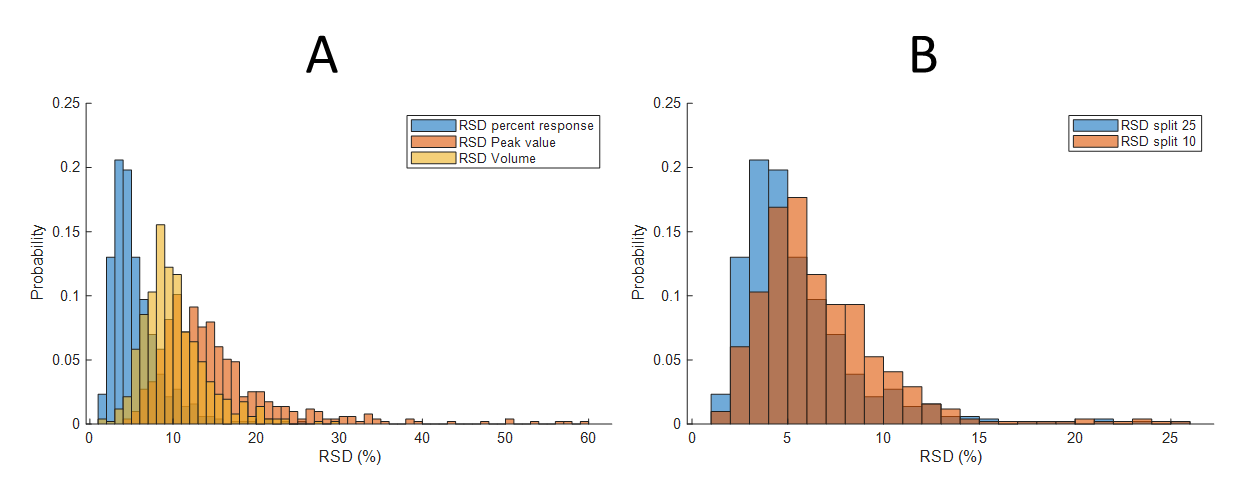
*Figure S1: Overview of results from repeatability (n=11) experiments. Distributions of (A): RSDs calculated from different intensity metrics of the method using a split ratio of 25:1; (B): RSDs from the obtained percentage response for the methods using a split ratio of 25:1 and 10:1.*

## Supplementary table 2 – Carryover results

*Table S2: Overview of tentatively identified compounds and their base peak m/z intensities monitored for 5 blank measurements following a measurement of the Laphroaig PX whisky.*

| **Compound** | **Laphroaig PX** | **Blank1** | **Blank2** | **Blank 3** | **Blank 4** | **Blank 5** | **Carryover1 (%)** | **Carryover 2 (%)** | **Carryover3 (%)** | **Carryover4 (%)** | **Carryover5 (%)** |
| --- | --- | --- | --- | --- | --- | --- | --- | --- | --- | --- | --- |
| Ethanamine, N-ethyl-N-nitroso- | 6785 | 0 | 47 | 0 | 0 | 0 | 0.0 | 0.7 | 0.0 | 0.0 | 0.0 |
| Hexanoic acid, ethyl ester | 14001 | 0 | 0 | 0 | 0 | 0 | 0.0 | 0.0 | 0.0 | 0.0 | 0.0 |
| 1-Butanol, 2-methyl-, (S)- | 1684033 | 150 | 454 | 354 | 253 | 280 | 0.0 | 0.0 | 0.0 | 0.0 | 0.0 |
| Phenol | 2142882 | 1223 | 484 | 909 | 400 | 319 | 0.1 | 0.0 | 0.0 | 0.0 | 0.0 |
| Phenol, 2-methoxy- | 738412 | 466 | 46 | 267 | 306 | 294 | 0.1 | 0.0 | 0.0 | 0.0 | 0.0 |
| Octanoic acid, ethyl ester | 82418 | 64 | 33 | 0 | 111 | 0 | 0.1 | 0.0 | 0.0 | 0.1 | 0.0 |
| Creosol | 260727 | 211 | 118 | 123 | 89 | 0 | 0.1 | 0.0 | 0.0 | 0.0 | 0.0 |
| p-Cresol | 1475018 | 1239 | 742 | 1149 | 841 | 469 | 0.1 | 0.1 | 0.1 | 0.1 | 0.0 |
| Phenol, 4-ethyl-2-methoxy- | 457230 | 557 | 172 | 376 | 0 | 0 | 0.1 | 0.0 | 0.1 | 0.0 | 0.0 |
| 1,3,5-Pentanetriol | 109748 | 174 | 264 | 417 | 69 | 326 | 0.2 | 0.2 | 0.4 | 0.1 | 0.3 |
| Phenol, 4-ethyl- | 1453061 | 2873 | 795 | 668 | 1117 | 271 | 0.2 | 0.1 | 0.0 | 0.1 | 0.0 |
| Phenol, 2-methyl- | 1220587 | 2627 | 489 | 478 | 244 | 139 | 0.2 | 0.0 | 0.0 | 0.0 | 0.0 |
| 2-Furancarboxaldehyde, 5-methyl- | 467100 | 1261 | 305 | 236 | 150 | 101 | 0.3 | 0.1 | 0.1 | 0.0 | 0.0 |
| Acetic acid, 2-phenylethyl ester | 611218 | 1880 | 865 | 888 | 948 | 395 | 0.3 | 0.1 | 0.1 | 0.2 | 0.1 |
| Phenylethyl Alcohol | 6972076 | 28993 | 10625 | 5897 | 4324 | 1134 | 0.4 | 0.2 | 0.1 | 0.1 | 0.0 |
| Decanoic acid, ethyl ester | 357319 | 1487 | 1266 | 41 | 0 | 13 | 0.4 | 0.4 | 0.0 | 0.0 | 0.0 |
| Glycerin | 9315999 | 44965 | 1659 | 195 | 156 | 336 | 0.5 | 0.0 | 0.0 | 0.0 | 0.0 |
| Pentadecanoic acid, 3-methylbutyl ester | 74011 | 542 | 523 | 52 | 204 | 341 | 0.7 | 0.7 | 0.1 | 0.3 | 0.5 |
| Octanoic acid | 698139 | 5134 | 2406 | 1138 | 615 | 488 | 0.7 | 0.3 | 0.2 | 0.1 | 0.1 |
| Butanedioic acid, diethyl ester | 1307033 | 11500 | 2094 | 144 | 409 | 41 | 0.9 | 0.2 | 0.0 | 0.0 | 0.0 |
| Palmitoleic acid ethyl ester | 137057 | 1417 | 586 | 138 | 370 | 0 | 1.0 | 0.4 | 0.1 | 0.3 | 0.0 |
| Dodecanoic acid, ethyl ester | 1087313 | 11407 | 3003 | 2216 | 1099 | 396 | 1.0 | 0.3 | 0.2 | 0.1 | 0.0 |
| Phenol, 2,6-dimethyl- | 945355 | 10147 | 3007 | 1532 | 1811 | 358 | 1.1 | 0.3 | 0.2 | 0.2 | 0.0 |
| Phenol, 2,3-dimethyl- | 32740 | 353 | 226 | 468 | 40 | 24 | 1.1 | 0.7 | 1.4 | 0.1 | 0.1 |
| Hexadecanoic acid, ethyl ester | 1528467 | 18292 | 8912 | 4338 | 2718 | 900 | 1.2 | 0.6 | 0.3 | 0.2 | 0.1 |
| Tetradecanoic acid, ethyl ester | 420869 | 5102 | 2212 | 843 | 99 | 41 | 1.2 | 0.5 | 0.2 | 0.0 | 0.0 |
| Furfural | 5667932 | 79642 | 22446 | 12466 | 6885 | 2698 | 1.4 | 0.4 | 0.2 | 0.1 | 0.0 |
| 2(3H)-Furanone, 5-butyldihydro-4-methyl- | 598290 | 9082 | 4792 | 2922 | 3272 | 338 | 1.5 | 0.8 | 0.5 | 0.5 | 0.1 |
| 1-Butanol, 3-methyl-, acetate | 98817 | 1540 | 1486 | 1479 | 1070 | 953 | 1.6 | 1.5 | 1.5 | 1.1 | 1.0 |
| Butanoic acid, 2-methyl- | 13716 | 219 | 469 | 12 | 129 | 162 | 1.6 | 3.4 | 0.1 | 0.9 | 1.2 |
| Hexanoic acid, 2-phenylethyl ester | 37663 | 605 | 917 | 900 | 260 | 351 | 1.6 | 2.4 | 2.4 | 0.7 | 0.9 |
| 1,3-Propanediol, 2-(hydroxymethyl)-2-nitro- | 5992480 | 107564 | 19928 | 7412 | 2700 | 1097 | 1.8 | 0.3 | 0.1 | 0.0 | 0.0 |
| 1-Tetradecanol | 352996 | 7144 | 3576 | 2167 | 1121 | 778 | 2.0 | 1.0 | 0.6 | 0.3 | 0.2 |
| Hexadecanol | 867326 | 21624 | 8488 | 6071 | 4201 | 2579 | 2.5 | 1.0 | 0.7 | 0.5 | 0.3 |
| n-Decanoic acid | 651564 | 17136 | 5417 | 3048 | 1907 | 612 | 2.6 | 0.8 | 0.5 | 0.3 | 0.1 |
| Tetradecanoic acid | 54424 | 1472 | 767 | 117 | 565 | 54 | 2.7 | 1.4 | 0.2 | 1.0 | 0.1 |
| Dodecanoic acid | 234975 | 6494 | 2224 | 858 | 1125 | 46 | 2.8 | 0.9 | 0.4 | 0.5 | 0.0 |
| Benzaldehyde, 3-hydroxy-4-methoxy- | 914050 | 26670 | 8460 | 4661 | 4021 | 1080 | 2.9 | 0.9 | 0.5 | 0.4 | 0.1 |
| Oxalic acid, heptyl 2-phenylethyl ester | 91096 | 2687 | 1453 | 959 | 894 | 519 | 2.9 | 1.6 | 1.1 | 1.0 | 0.6 |
| Decanoic acid, 2-phenylethyl ester | 370320 | 12113 | 5479 | 3624 | 3939 | 1694 | 3.3 | 1.5 | 1.0 | 1.1 | 0.5 |
| Butanoic acid, 3-methyl- | 33821 | 1185 | 571 | 183 | 442 | 182 | 3.5 | 1.7 | 0.5 | 1.3 | 0.5 |
| Apocynin | 46540 | 1928 | 802 | 524 | 63 | 0 | 4.1 | 1.7 | 1.1 | 0.1 | 0.0 |
| Unknown | 219285 | 9517 | 3776 | 629 | 455 | 375 | 4.3 | 1.7 | 0.3 | 0.2 | 0.2 |
| Benzaldehyde, 4-hydroxy-3,5-dimethoxy- | 2480677 | 117782 | 39464 | 21515 | 13990 | 5375 | 4.7 | 1.6 | 0.9 | 0.6 | 0.2 |
| Linoleic acid ethyl ester | 84022 | 4836 | 4185 | 4218 | 3736 | 2707 | 5.8 | 5.0 | 5.0 | 4.4 | 3.2 |
| Ethanone, 1-(4-hydroxy-3,5-dimethoxyphenyl)- | 81904 | 5155 | 1629 | 1427 | 802 | 0 | 6.3 | 2.0 | 1.7 | 1.0 | 0.0 |
| Palmitoleic acid | 36057 | 2355 | 1153 | 1836 | 1018 | 880 | 6.5 | 3.2 | 5.1 | 2.8 | 2.4 |
| Coniferyl aldehyde | 296648 | 20863 | 6108 | 2273 | 1586 | 15 | 7.0 | 2.1 | 0.8 | 0.5 | 0.0 |
| n-Hexadecanoic acid | 62390 | 4427 | 3028 | 2213 | 1883 | 551 | 7.1 | 4.9 | 3.5 | 3.0 | 0.9 |
| β-D-Glucopyranose, 1,6-anhydro- | 1830933 | 142692 | 58970 | 36234 | 28288 | 15866 | 7.8 | 3.2 | 2.0 | 1.5 | 0.9 |
| 3-Deoxy-d-mannoic lactone | 1106476 | 102021 | 34085 | 11068 | 5551 | 255 | 9.2 | 3.1 | 1.0 | 0.5 | 0.0 |
| Inositol, 1-deoxy- | 5440243 | 521490 | 199696 | 113710 | 71594 | 31314 | 9.6 | 3.7 | 2.1 | 1.3 | 0.6 |
| 5-Hydroxymethylfurfural | 9148702 | 1044187 | 191623 | 67869 | 25411 | 153 | 11.4 | 2.1 | 0.7 | 0.3 | 0.0 |
| 3,5-Dimethoxy-4-hydroxycinnamaldehyde | 153867 | 19394 | 5727 | 5810 | 1915 | 211 | 12.6 | 3.7 | 3.8 | 1.2 | 0.1 |
| Homosyringaldehyde | 73241 | 11839 | 6059 | 2806 | 1726 | 682 | 16.2 | 8.3 | 3.8 | 2.4 | 0.9 |
| trans-Sinapyl alcohol | 53873 | 9712 | 4358 | 2841 | 1889 | 925 | 18.0 | 8.1 | 5.3 | 3.5 | 1.7 |

## Supplementary figure 2 – Liner Inspection


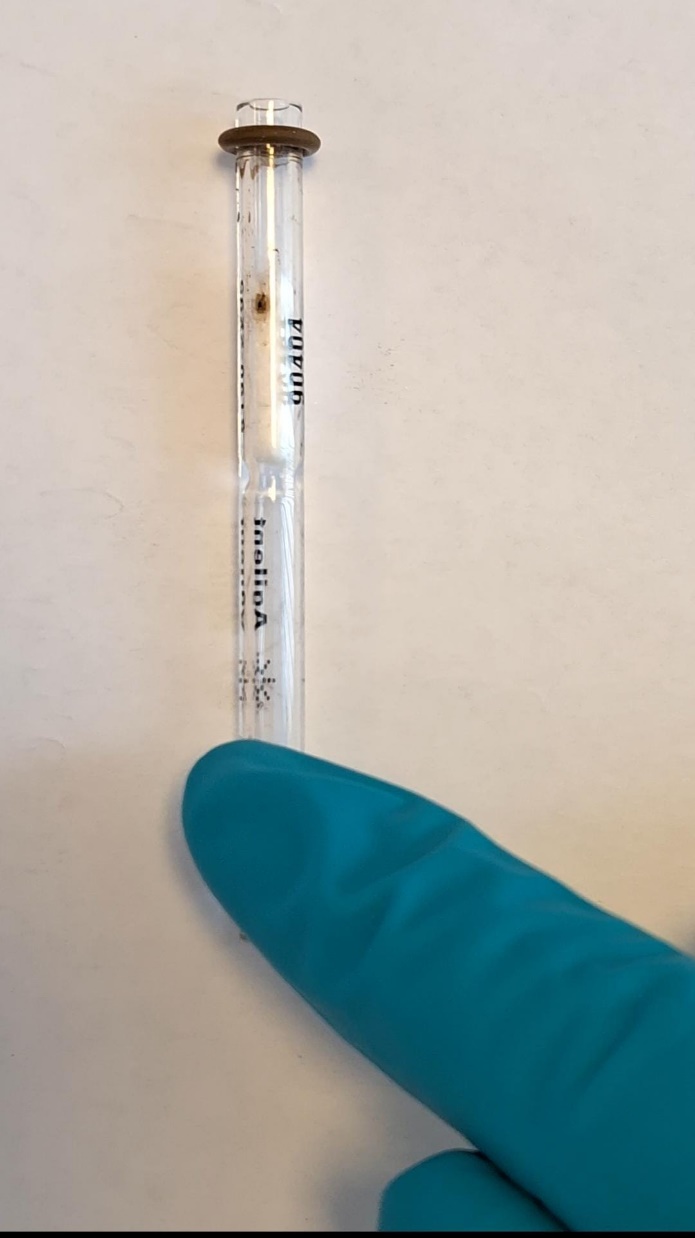

*Figure S2: photograph of the liner after all major sample sequences had been performed.*

## Supplementary figure 3 – RSD overview for all samples


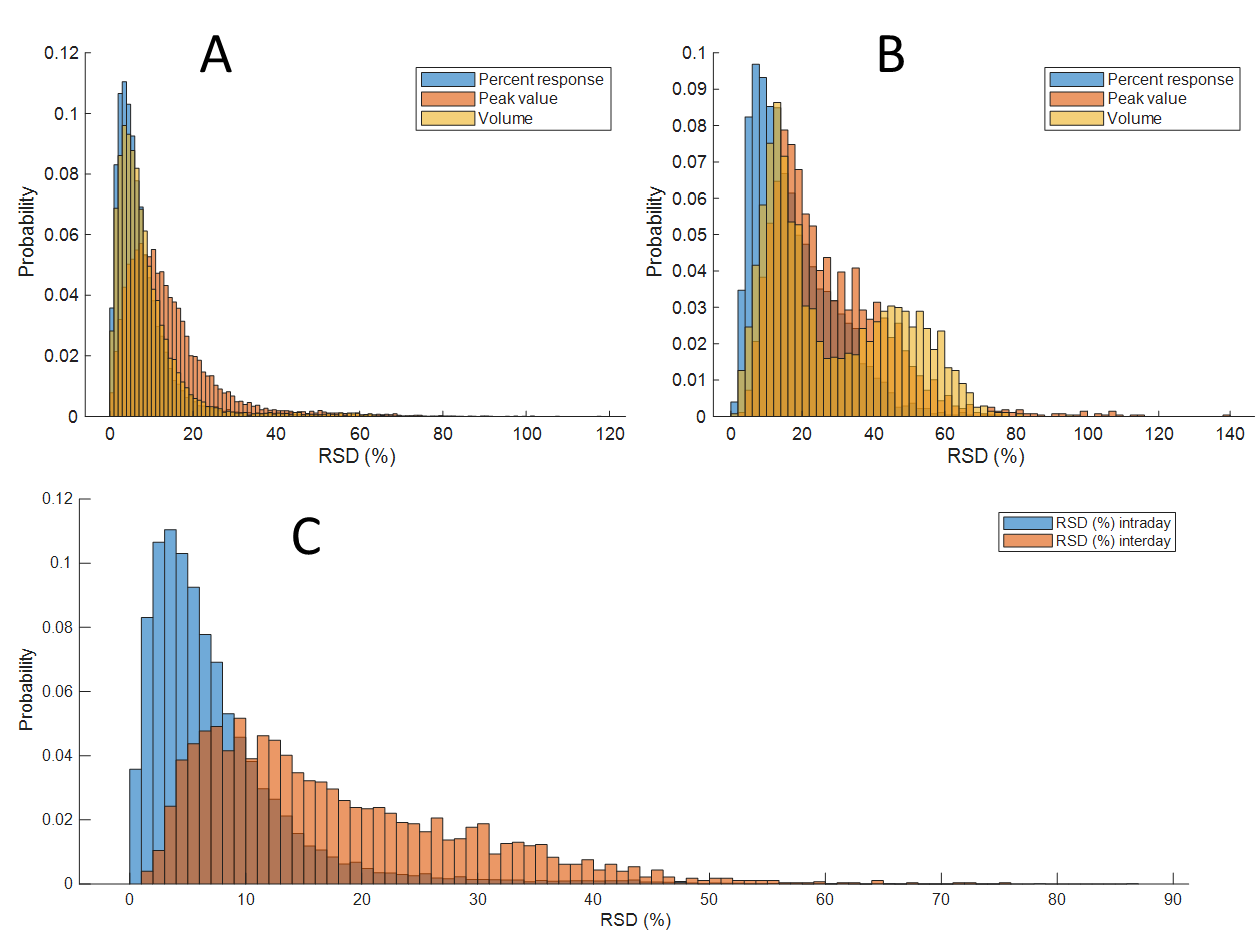
*Figure S3: RSD distributions of different peak-intensity metrics, calculated from (A): percent response, peak value, and integrated volume (in blue, orange, and yellow, respectively) using only the triplicate samples and excluding the samples measured over different sequences; (B): percent response, peak value, and integrated volume (in blue, orange, and yellow, respectively) using only the samples that were measured over different sequences; (C): the percentage response of both only the triplicate samples (in blue) and the samples that were measured over different sequences (orange).*

## Supplementary figure 4 – PCA including blank measurements

*Figure S4: Principal component analysis of the measured whiskies and the blank measurements. Several of the samples that were measured over multiple sequences have been outlined with their respective color.*

## Supplementary figure 5 – Samples PCA including the simulated adulteration

*Figure S5: Principal component analysis of only the samples and excluding the blank measurements. The x-axis has been cropped for better visibility of the simulated adulteration denoted by the red dot, removing two datapoints of the Laphroaig PX whisky from view.*
